# Association of cheese and yogurt intake with sleep duration in preschool-aged children: a 6-month prospective cohort study

**DOI:** 10.3389/fnut.2025.1685564

**Published:** 2026-01-05

**Authors:** Cuilan Lin, Zhuling Yang, Yawen Yuan, Xin Lai, Simao Fu, Dongxue Pan

**Affiliations:** 1 The Second School of Clinical Medicine, Southern Medical University, Guangzhou, Guangdong, China; 2 Bo’ai Hospital of Zhongshan, Zhongshan, Guangdong, China; 3 Zhongshan People’s Hospital, Zhongshan, Guangdong, China; 4 Shenzhen Hospital, The Chinese University of Hong Kong, Shenzhen, Guangdong, China

**Keywords:** preschool-aged children, cheese intake, yogurt intake, sleep duration, probiotic dairy

## Abstract

**Objective:**

To evaluate whether baseline cheese and yogurt intake is associated with sleep duration at baseline, 3- and 6-month follow-ups among preschool-aged children.

**Methods:**

We conducted a prospective cohort study in Zhongshan City, Guangdong Province, China. Parents completed baseline questionnaires on cheese and yogurt intake frequency, sleep duration and potential confounders, with sleep time followed up at 3 and 6 months. Sleep duration of < 10 h per day was defined as insufficient. Multivariable logistic regression and mixed-effects models were used to evaluate the association between the baseline cheese and yogurt intake and sleep duration at baseline, 3- and 6-month follow-ups.

**Results:**

A total of 221 preschool-aged children were included in the analysis. No significant association was found between yogurt consumption and sleep duration at any time point. For cheese intake, a significant trend was identified at 6 months (*p* = 0.007), and cheese intake ≥ 7 servings/week showed a reduced prevalence of insufficient sleep (adjusted OR = 0.001, 99.2% CI: 0.000–0.168). Mixed-effects models confirmed a significant interaction between high cheese intake and 6-month follow-up (OR = 0.217, 95% CI: 0.052–0.917).

**Conclusion:**

Our findings suggest that frequent cheese intake may reduce the risk of insufficient sleep in preschool-aged children, whereas yogurt shows no comparable association. These differential results underscore the need for product-specific analyses. Despite limitations in sample size and measurement, the study adds to evidence linking diet, microbiota, and sleep. Further research should clarify underlying mechanisms and guide actionable, child-appropriate dietary recommendations.

## Introduction

Adequate sleep in preschool-aged children is a critical determinant of neurodevelopment, metabolic health, and long-term wellbeing. Evidence consistently associates insufficient sleep in this age group with a range of adverse outcomes, for example, disrupting the physiological sleep rhythm can have long-term negative effects on motor, cognitive, language, and emotional development (1–3), and increasing the risk of obesity (4) and metabolic disorders (5), as well as potential impairment of antioxidant capacity (6). Given the multisystem, enduring impact of insufficient sleep, identifying modifiable risk factors early in life is a public-health priority.

Probiotic-containing and other fermented foods are increasingly studied for potential health benefits, particularly through modulation of the gut microbiota (7, 8). The gut microbiota, as a complex and dynamic ecosystem within the human body, is closely linked to immune modulation, metabolic functions, and neurological behaviors. Research indicates that probiotic intake can significantly enhance the α-diversity of the gut microbiome (9, 10) and alter the single nucleotide variants, growth rates, and network interactions of the indigenous microbiota through rapid evolutionary responses (9). Meanwhile, studies suggest that the gut microbiota regulates sleep through the microbiome-gut-brain axis (11–13). In adult studies, Liu et al. reported that *Bifidobacterium longum* BLa80 significantly improved sleep quality in healthy adults by alleviating gut dysbiosis (14). Yang et al. found that the intake of probiotic-containing dairy products (such as yogurt) was associated with a reduced risk of sleep disorders in U.S. adults, especially among males, white individuals, and those with normal BMI (15). Greater microbial diversity has also been linked to better sleep efficiency (16), longer total sleep time (17), and less sleep fragmentation (16). However, research on children is limited. A large-scale birth cohort study from the Japan Environment and Children’s Study (JECS) indicated that maternal intake of fermented foods during pregnancy might positively influence the duration of sleep in early childhood (18), with a significant but limited association between maternal intake of fermented foods during pregnancy and longer sleep duration at 3 years (19). Additionally, this cohort study found that there was no association between the intake of yogurt or cheese at 1 year of age and sleep duration at the same age, but suggested that early yogurt intake might have a positive effect on sleep at age 3 years, whereas cheese intake showed no such effect (20). Currently, to the best of our knowledge, no studies have investigated the relationship between the intake of probiotic-containing dairy products (e.g., cheese and yogurt) and sleep in preschool-aged children.

In this study, our research was mainly to explore the effects of baseline cheese and yogurt intake on the sleep at baseline, 3-month-up and 6-month follow-up in preschool-aged children.

## Materials and methods

### Subjects and procedure

**FIGURE 1:**
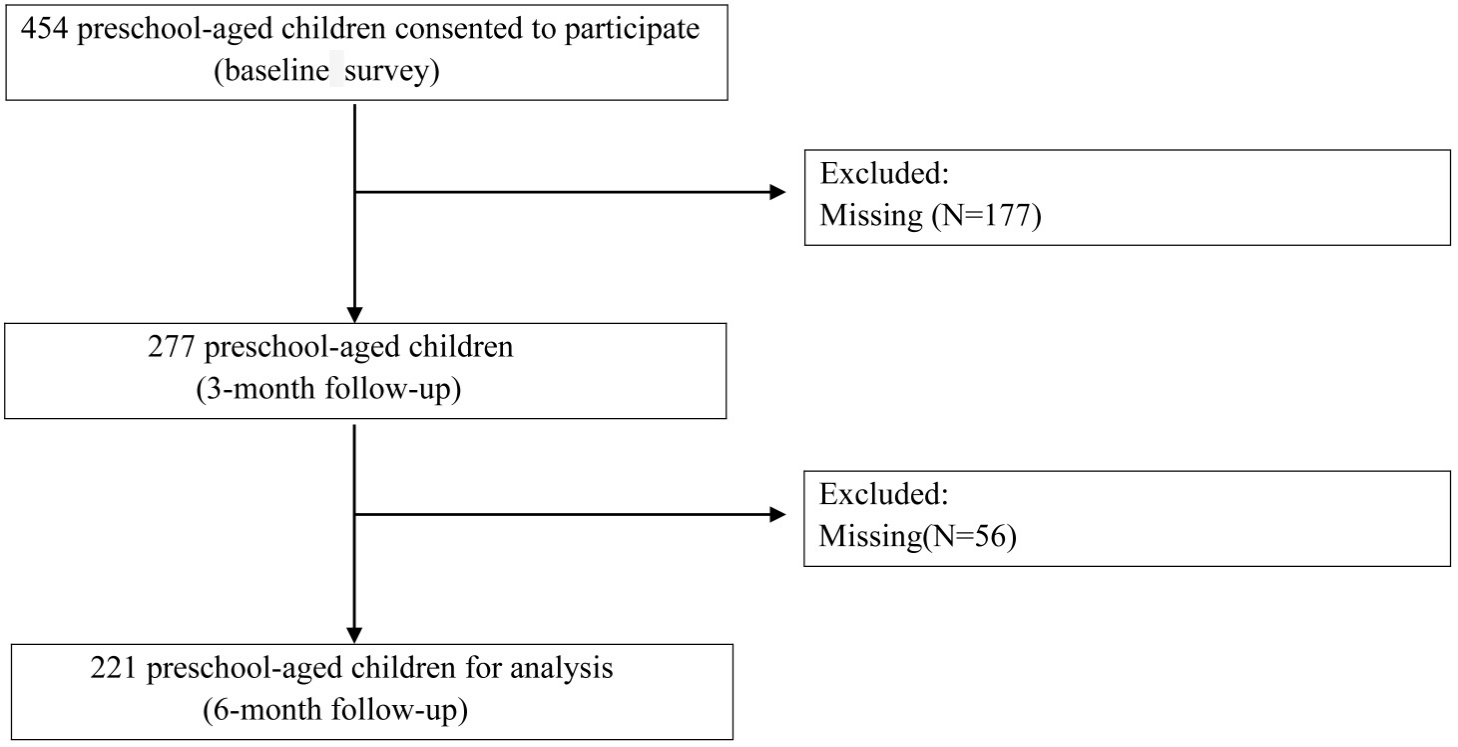
Flowchart outlining the participant recruitment and exclusion criteria.

This study adopted a prospective cohort design to investigate the relationship between cheese intake and yogurt intake and sleep time in preschool-aged children. Between March and June 2023, recruitment information was randomly posted in the WeChat groups of kindergartens in Zhongshan City, Guangdong Province, China. After obtaining consent from the guardians, the informed consent form was signed, and an online baseline survey was conducted, the survey context was as follows: sex, birth conditions, parental education level, annual household income, postnatal feeding method, lactose intolerance, regular calcium supplementation, regular vitamin D supplementation, fresh milk intake, cheese intake, yogurt intake, red meat intake, processed meat, snack intake, sugar-sweetened beverages intake, frequency of exercise, total sleep time per day, bedtime per night at the baseline, 3-month follow-up and 6-month follow-up. Follow-up assessments for sleep time were conducted at 3 and 6 months (Figure 1). Inclusion criteria: (1) Healthy children aged ≥ 3 and ≤ 6 years, regardless of sex; (2) Willingness to sign the informed consent form and ability to comply with the study protocol for follow-up. Exclusion criteria: (1) Children with milk allergies or other conditions that contraindicate the consumption of dairy products; (2) Children with severe heart, liver, kidney, blood, digestive, or neurological diseases; (3) Children currently or recently (within the past 6 months) involved in any clinical studies. Withdrawal criteria: (1) Refusal to continue follow-up after signing the informed consent form; (2) Inability to adhere to the study protocol; (3) Adverse events related to the study; (4) Any other circumstances that render continued participation inappropriate. The study protocol was approved by the institutional review boards of the Shanghai Children’s Medical Center, Shanghai Jiao Tong University School of Medicine and BoAi Hospital of Zhongshan.

To assess the frequency of cheese intake and yogurt intake, guardians of the participants completed an online questionnaire. The survey included the following items: In the past 3 months, how often has your child consumed cheese? (Response options: Never; 1 serving /week; 2–6 servings/week; ≥ 7 servings/week); In the past 3 months, how often has your child consumed yogurt? (Response options: Never; 1 serving /week; 2–6 servings/week; ≥ 7 servings/week).

### Outcome categories

To assess sleep time at baseline, 3-month follow-up, and 6-month follow-up, parents were asked to report their child’s total sleep time per day in the questionnaire. Participants were categorized into quartiles based on the intake levels of cheese and yogurt to evaluate their risk of insufficient sleep duration. According to the National Sleep Foundation, the recommended total sleep duration for preschool-aged children is 1–13 h per day. Therefore, insufficient sleep duration was defined as sleep time < 10 h in children aged 3–6 years (21).

### Confounders in logistic regression analysis

The following confounding variables were adjusted for in the multivariable logistic regression analysis: sex, birth conditions, parental education level, annual household income, postnatal feeding method, lactose intolerance, regular calcium supplementation, regular vitamin D supplementation, fresh milk intake, cheese intake/ yogurt intake, red meat intake, processed meat, snack intake, sugar-sweetened beverages intake, frequency of exercise, bedtime per night at the baseline/bedtime per night at the 3-month follow-up/ bedtime per night at the 6-month follow-up.

### Statistical analysis

Group characteristics across different yogurt or cheese intake levels were summarized and compared using the Chi-square test, while Fisher’s exact test was applied when any expected cell frequency in the contingency table was ≤ 5. Univariate and multivariate logistic regression analyses were performed to examine the association between baseline cheese intake frequency (or yogurt intake frequency) and the incidence of insufficient sleep duration ( < 10 h) at baseline, 3-month follow-up, and 6-month follow-up. Both crude and adjusted odds ratios (ORs) with confidence intervals (CIs) were calculated. Adjusted ORs were computed with adjusting for the covariates (confounders), while crude ORs were calculated without adjusting for these covariates. Trend tests were conducted by treating the ordinal intake levels for cheese and yogurt as continuous variables in multivariate logistic models. The Bonferroni correction was applied for both the trend tests and calculating the confidence intervals of ORs.

For models where the corrected OR confidence interval upper limit was below 1, a *post-hoc* power analysis was conducted to assess the adequacy of the sample size. Specifically, we performed bootstrap resampling to generate samples of the same size as the analytical dataset. Based on the model estimates, the outcome variable was simulated 1,000 times. The proportion of these simulations in which the adjusted OR upper confidence limit remained < 1 and the adjusted trend test *p*-value was < 0.05 was then the *post-hoc* statistical power.

In addition, we fitted a mixed-effects logistic regression model to analyze the association of cheese intake and yogurt intake with sleep duration at the three different time points, which random intercepts to account for the correlation within subjects. This model included fixed effects of time, yogurt intake, cheese intake, and the interaction terms between each of the consumption variables time. To mitigate potential model convergence issues due to the complexity of the model, we dichotomized the consumption variables. Specifically, we categorized “never” and “1 serving/week” as low consumption and “2–6 servings/week” and “≥ 7 servings/week” as high consumption.

All statistical analyses were performed using the R version 4.0.4. We established statistical significance at a two-sided *p*-value < 0.05.

Bold indicates significance.

**^*α*^**CI after application of Bonferroni correction corresponding to the 99.2% ( = 100–5/6) CIs.

**^*β*^**Adjusted for Sex, Birth conditions, Parental education level, Annual household income, Postnatal feeding method, Lactose intolerance, Regular calcium supplementation, Regular vitamin D supplementation, Fresh milk intake, Cheese intake/ Yogurt intake, Red meat intake, Processed meat, Snack intake, Sugar-sweetened beverages intake, Frequency of exercise, Bedtime per night at the baseline/Bedtime per night at the 3-month follow-up/ Bedtime per night at the 6-month follow-up.

**^*δ*^**Values were multiplied by 6 so that the significance level after Bonferroni correction remains at 5%.

## Results

The baseline characteristics of the participants (*n* = 221) are presented in Tables 1, 2. Parental education level, yogurt intake, processed meat intake, and snack intake were significantly different across the groups of cheese consumption frequency in Table 1. Parental education level, postnatal feeding method, fresh milk intake, cheese intake, snack intake, sugar-sweetened beverages intake and frequency of exercise were significantly different across the groups of yogurt consumption frequency in Table 2. In addition, in Supplementary Table 1, we compared the baseline characteristics of the complete (*n* = 221) and missing data (*n* = 233), significant differences between the two groups were observed in annual household income, postnatal feeding method and snack intake, whereas no significant differences were found in sleep duration, yogurt intake and cheese intake. The above differences in baseline characteristics necessitate the use of a multivariable logistic model for covariate adjustment.

**TABLE 1:** The characteristics of participant cheese consumption frequency at baseline.

Cheese consumption at baseline					
Characteristics	Never (*n*, %)	1 serving/week (*n*, %)	2–6 servings/ week (*n*, %)	≥ 7 servings/ week (*n*, %)	*p*-value
**Sex**					0.435
Boy	45 (60.8%)	55 (50.9%)	17 (58.6%)	4 (40%)	
Girl	29 (39.2%)	53 (49.1%)	12 (41.4%)	6 (60%)	
**Birth conditions**					0.547
Preterm infants	7 (9.5%)	7 (6.5%)	3 (10.3%)	2 (20%)	
Term small for gestational age infants	3 (4.1%)	3 (2.8%)	2 (6.9%)	0 (0%)	
Term normal birth weight infants	63 (85.1%)	98 (90.7%)	24 (82.8%)	8 (80%)	
Large for gestational age infants	1 (1.4%)	0 (0%)	0 (0%)	0 (0%)	
**Parental education level**					**0.005**
High school or below	11 (14.9%)	22 (20.4%)	5 (17.2%)	4 (40%)	
University	63 (85.1%)	82 (75.9%)	21 (72.4%)	4 (40%)	
Graduate school or above	0 (0%)	4 (3.7%)	3 (10.3%)	2 (20%)	
**Annual household income (CNY)**					0.106
≤ 8,000	16 (21.6%)	21 (19.4%)	4 (13.8%)	3 (30%)	
8,001–15,000	44 (59.5%)	57 (52.8%)	10 (34.5%)	4 (40%)	
15,001–30,000	12 (16.2%)	21 (19.4%)	11 (37.9%)	3 (30%)	
≥ 30,001	2 (2.7%)	9 (8.3%)	4 (13.8%)	0 (0%)	
**Postnatal feeding method**					0.368
≥ 6 months exclusive breastfeeding	38 (51.4%)	65 (60.2%)	13 (44.8%)	5 (50%)	
< 6 months exclusive breastfeeding	11 (14.9%)	10 (9.3%)	3 (10.3%)	2 (20%)	
Mixed feeding	21 (28.4%)	28 (25.9%)	10 (34.5%)	1 (10%)	
Exclusive formula feeding	4 (5.4%)	5 (4.6%)	3 (10.3%)	2 (20%)	
**Lactose intolerance**					0.423
No	73 (98.6%)	106 (98.1%)	27 (93.1%)	10 (100%)	
Yes*	1 (1.4%)	2 (1.9%)	2 (6.9%)	0 (0%)	
**Regular calcium supplementation**					0.918
No	61 (82.4%)	86 (79.6%)	23 (79.3%)	9 (90%)	
Yes	13 (17.6%)	22 (20.4%)	6 (20.7%)	1 (10%)	
**Regular vitamin D supplementation**					0.316
No	52 (70.3%)	72 (66.7%)	17 (58.6%)	9 (90%)	
Yes	22 (29.7%)	36 (33.3%)	12 (41.4%)	1 (10%)	
**Fresh milk intake**					0.528
Never	7 (9.5%)	8 (7.4%)	0 (0%)	0 (0%)	
1 serving/week	13 (17.6%)	16 (14.8%)	2 (6.9%)	2 (20%)	
2–6 servings/week	20 (27%)	31 (28.7%)	11 (37.9%)	1 (10%)	
≥ 7 servings/week	34 (45.9%)	53 (49.1%)	16 (55.2%)	7 (70%)	
**Yogurt intake**					** < 0.001**
Never	24 (32.4%)	6 (5.6%)	1 (3.4%)	0 (0%)	
1 serving/week	33 (44.6%)	57 (52.8%)	2 (6.9%)	2 (20%)	
2–6 servings/week	13 (17.6%)	31 (28.7%)	20 (69%)	2 (20%)	
≥ 7 servings/week	4 (5.4%)	14 (13%)	6 (20.7%)	6 (60%)	
**Red meat intake^#^**					0.305
Never	16 (21.6%)	13 (12%)	1 (3.4%)	0 (0%)	
1 serving/week	20 (27%)	37 (34.3%)	10 (34.5%)	4 (40%)	
2–6 servings/week	24 (32.4%)	36 (33.3%)	12 (41.4%)	2 (20%)	
≥ 7 servings/week	14 (18.9%)	22 (20.4%)	6 (20.7%)	4 (40%)	
**Processed meat^&^**					** < 0.001**
Never	52 (70.3%)	55 (50.9%)	14 (48.3%)	5 (50%)	
1 serving/week	17 (23%)	50 (46.3%)	8 (27.6%)	3 (30%)	
2–6 servings/week	4 (5.4%)	2 (1.9%)	6 (20.7%)	0 (0%)	
≥ 7 servings/week	1 (1.4%)	1 (0.9%)	1 (3.4%)	2 (20%)	
**Snack intake^Δ^**					**0.003**
Never	14 (18.9%)	5 (4.6%)	0 (0%)	1 (10%)	
1 serving/week	38 (51.4%)	61 (56.5%)	12 (41.4%)	5 (50%)	
2–6 servings/week	20 (27%)	37 (34.3%)	13 (44.8%)	2 (20%)	
≥ 7 servings/week	2 (2.7%)	5 (4.6%)	4 (13.8%)	2 (20%)	
**Sugar-sweetened beverages intake**					0.33
Never	27 (36.5%)	35 (32.4%)	10 (34.5%)	5 (50%)	
1 serving/week	39 (52.7%)	60 (55.6%)	12 (41.4%)	3 (30%)	
2–6 servings/week	7 (9.5%)	13 (12%)	6 (20.7%)	2 (20%)	
≥ 7 servings/week	1 (1.4%)	0 (0%)	1 (3.4%)	0 (0%)	
**Frequency of exercise**					0.589
0–1 time/week	5 (6.8%)	5 (4.6%)	0 (0%)	2 (20%)	
2–3 times/week	25 (33.8%)	30 (27.8%)	8 (27.6%)	2 (20%)	
4–5 times/week	23 (31.1%)	42 (38.9%)	11 (37.9%)	4 (40%)	
6–7 times/week	21 (28.4%)	31 (28.7%)	10 (34.5%)	2 (20%)	

CNY, Chinese Yuan. *Unable to drink milk on an empty stomach, but can consume yogurt or cheese.

^#^Red meat, such as pork, beef, lamb, liver, blood products, etc.

^&^Red sausage, sausage, bacon, smoked meat, etc.

^Δ^Candy, jelly, ice cream, cake, cookies, chocolate, potato chips, etc. Bold indicates significance.

**TABLE 2:** The characteristics of participant yogurt consumption frequency at baseline.

Yogurt consumption at baseline					
Characteristics	Never (*n*, %)	1 serving/week (*n*, %)	2–6 servings/ week (*n*, %)	≥ 7 servings/ week (*n*, %)	*p*-value
**Sex**					0.512
Boy	16 (51.6%)	53 (56.4%)	39 (59.1%)	13 (43.3%)	
Girl	15 (48.4%)	41 (43.6%)	27 (40.9%)	17 (56.7%)	
**Birth conditions**					0.663
Preterm infants	3 (9.7%)	6 (6.4%)	8 (12.1%)	2 (6.7%)	
Term small for gestational age infants	2 (6.5%)	3 (3.2%)	3 (4.5%)	0 (0%)	
Term normal birth weight infants	26 (83.9%)	85 (90.4%)	54 (81.8%)	28 (93.3%)	
Large for gestational age infants	0 (0%)	0 (0%)	1 (1.5%)	0 (0%)	
**Parental education level**					**0.001**
High school or below	3 (9.7%)	22 (23.4%)	13 (19.7%)	4 (13.3%)	
University	26 (83.9%)	72 (76.6%)	52 (78.8%)	20 (66.7%)	
Graduate school or above	2 (6.5%)	0 (0%)	1 (1.5%)	6 (20%)	
**Annual household income (CNY)**					0.262
≤ 8,000	6 (19.4%)	23 (24.5%)	13 (19.7%)	2 (6.7%)	
8,001–15,000	17 (54.8%)	51 (54.3%)	32 (48.5%)	15 (50%)	
15,001–30,000	7 (22.6%)	16 (17%)	16 (24.2%)	8 (26.7%)	
≥ 30,001	1 (3.2%)	4 (4.3%)	5 (7.6%)	5 (16.7%)	
**Postnatal feeding method**					**0.04**
≥ 6 months exclusive breastfeeding	17 (54.8%)	53 (56.4%)	30 (45.5%)	21 (70%)	
< 6 months exclusive breastfeeding	3 (9.7%)	13 (13.8%)	4 (6.1%)	6 (20%)	
Mixed feeding	10 (32.3%)	22 (23.4%)	25 (37.9%)	3 (10%)	
Exclusive formula feeding	1 (3.2%)	6 (6.4%)	7 (10.6%)	0 (0%)	
**Lactose intolerance**					0.356
No	31 (100%)	92 (97.9%)	65 (98.5%)	28 (93.3%)	
Yes*	0 (0%)	2 (2.1%)	1 (1.5%)	2 (6.7%)	
**Regular calcium supplementation**					0.575
No	23 (74.2%)	77 (81.9%)	56 (84.8%)	23 (76.7%)	
Yes	8 (25.8%)	17 (18.1%)	10 (15.2%)	7 (23.3%)	
**Regular vitamin D supplementation**					0.558
No	19 (61.3%)	67 (71.3%)	46 (69.7%)	18 (60%)	
Yes	12 (38.7%)	27 (28.7%)	20 (30.3%)	12 (40%)	
**Fresh milk intake**					** < 0.001**
Never	4 (12.9%)	6 (6.4%)	3 (4.5%)	2 (6.7%)	
1 serving/week	2 (6.5%)	25 (26.6%)	2 (3%)	4 (13.3%)	
2–6 servings/week	9 (29%)	20 (21.3%)	33 (50%)	1 (3.3%)	
≥ 7 servings/week	16 (51.6%)	43 (45.7%)	28 (42.4%)	23 (76.7%)	
**Cheese intake**					** < 0.001**
Never	24 (77.4%)	33 (35.1%)	13 (19.7%)	4 (13.3%)	
1 serving/week	6 (19.4%)	57 (60.6%)	31 (47%)	14 (46.7%)	
2–6 servings/week	1 (3.2%)	2 (2.1%)	20 (30.3%)	6 (20%)	
≥ 7 servings/week	0 (0%)	2 (2.1%)	2 (3%)	6 (20%)	
**Red meat intake^#^**					0.224
Never	7 (22.6%)	15 (16%)	4 (6.1%)	4 (13.3%)	
1 serving/week	10 (32.3%)	29 (30.9%)	21 (31.8%)	11 (36.7%)	
2–6 servings/week	10 (32.3%)	29 (30.9%)	29 (43.9%)	6 (20%)	
≥ 7 servings/week	4 (12.9%)	21 (22.3%)	12 (18.2%)	9 (30%)	
**Processed meat^&^**					0.137
Never	22 (71%)	54 (57.4%)	33 (50%)	17 (56.7%)	
1 serving/week	7 (22.6%)	37 (39.4%)	24 (36.4%)	10 (33.3%)	
2–6 servings/week	2 (6.5%)	2 (2.1%)	7 (10.6%)	1 (3.3%)	
≥ 7 servings/week	0 (0%)	1 (1.1%)	2 (3%)	2 (6.7%)	
**Snack intake ^Δ^**					**0.014**
Never	8 (25.8%)	6 (6.4%)	2 (3%)	4 (13.3%)	
1 serving/week	14 (45.2%)	58 (61.7%)	30 (45.5%)	14 (46.7%)	
2–6 servings/week	8 (25.8%)	27 (28.7%)	28 (42.4%)	9 (30%)	
≥ 7 servings/week	1 (3.2%)	3 (3.2%)	6 (9.1%)	3 (10%)	
**Sugar-sweetened beverages intake**					**0.014**
Never	14 (45.2%)	36 (38.3%)	16 (24.2%)	11 (36.7%)	
1 serving/week	14 (45.2%)	53 (56.4%)	33 (50%)	14 (46.7%)	
2–6 servings/week	3 (9.7%)	4 (4.3%)	16 (24.2%)	5 (16.7%)	
≥ 7 servings/week	0 (0%)	1 (1.1%)	1 (1.5%)	0 (0%)	
**Frequency of exercise**					**0.025**
0–1 time/week	2 (6.5%)	5 (5.3%)	2 (3%)	3 (10%)	
2–3 times/week	12 (38.7%)	32 (34%)	19 (28.8%)	2 (6.7%)	
4–5 times/week	6 (19.4%)	36 (38.3%)	22 (33.3%)	16 (53.3%)	
6–7 times/week	11 (35.5%)	21 (22.3%)	23 (34.8%)	9 (30%)	

*Unable to drink milk on an empty stomach, but can consume yogurt or cheese.

^#^Red meat, such as pork, beef, lamb, liver, blood products, etc.

^&^Red sausage, sausage, bacon, smoked meat, etc.

^Δ^Candy, jelly, ice cream, cake, cookies, chocolate, potato chips, etc. Bold indicates significance.

Table 3 presented the unadjusted and adjusted odds ratios (ORs) with confidence intervals (CIs) corrected by the Bonferroni approach for the association between insufficient sleep and consumption of cheese and yogurt at baseline, 3-month follow-up, and 6-month follow-up. The variance inflation factors (VIF) for all variables in the adjusted models were below 1.6, suggesting the absence of severe multicollinearity. No significant differences were observed between the yogurt intake and sleep time at the three different time points, both before and after adjusting for potential confounders. Regarding cheese intake and sleep time, no significant association was observed at baseline and 3-month follow-up. However, at the 6-month follow-up, the trend test was significant (*p* = 0.007), and cheese intake ≥ 7 servings/week showed a reduced prevalence of insufficient sleep. To further validate the primary results, a mixed-effects logistic regression model with random intercepts was fitted. The association with higher cheese intake remained statistically significant at the 6-month follow-up (Supplementary Table 2), consistent with the findings presented in Table 3.

**TABLE 3:** The associations between cheese intake and yogurt intake and sleep time at the baseline, 3-month follow-up, and 6-month follow-up.

Baseline	Sleep time ≥ 10 h	Sleep time < 10 h	Crude model^*α*^ Odds ratios (99.2% CI)	Adjusted model^α^,^β^ Odds ratios (99.2% CI)	*p*-value for trend^*δ*^
**Yogurt intake**					
Never	18	13	Ref	Ref	3.012
1 serving/week	50	44	1.218 (0.406, 3.795)	1.463 (0.278, 7.868)	
2–6 servings/week	30	36	1.662 (0.524, 5.474)	1.721 (0.286, 10.574)	
≥ 7 servings/week	22	8	0.503 (0.110, 2.108)	0.523 (0.063, 4.029)	
**Cheese intake**					
Never	38	36	Ref	Ref	1.852
1 serving/week	63	45	0.754 (0.337, 1.681)	0.859 (0.256, 2.868)	
2–6 servings/week	12	17	1.495 (0.468, 4.988)	3.227 (0.494, 23.402)	
≥ 7 servings/week	7	3	0.452 (0.047, 2.804)	1.214 (0.024, 42.318)	
**3-month follow-up**					
**Yogurt intake**					
Never	13	18	Ref	Ref	0.864
1 serving/week	36	58	1.164 (0.372, 3.525)	3.702 (0.497, 34.421)	
2–6 servings/week	24	42	1.264 (0.382, 4.095)	2.667 (0.293, 29.481)	
≥ 7 servings/week	11	19	1.247 (0.311, 5.129)	7.341 (0.563, 123.668)	
**Cheese intake**					
Never	25	49	Ref	Ref	1.679
1 serving/week	42	66	0.802 (0.344, 1.831)	0.576 (0.139, 2.262)	
2–6 servings/week	10	19	0.969 (0.292, 3.452)	1.35 (0.132, 13.522)	
≥ 7 servings/week	7	3	0.219 (0.023, 1.368)	0.096 (0.001, 2.741)	
**6-month follow-up**					
**Yogurt intake**					
Never	6	25	Ref	Ref	0.162
1 serving/week	31	63	0.488 (0.110, 1.697)	0.44 (0.026, 5.635)	
2–6 servings/week	11	55	1.2 (0.243, 5.127)	2.842 (0.160, 49.027)	
≥ 7 servings/week	11	19	0.415 (0.078, 1.905)	3.974 (0.112, 146.695)	
**Cheese intake**					
Never	14	60	Ref	Ref	**0.007**
1 serving/week	32	76	0.554 (0.202, 1.411)	0.344 (0.051, 1.934)	
2–6 servings/week	6	23	0.894 (0.222, 4.292)	0.088 (0.004, 1.374)	
≥ 7 servings/week	7	3	**0.100 (0.010, 0.654)**	**0.001 (0.000, 0.168)**	

Bold indicates significance. ^α^CI after application of Bonferroni correction corresponding to the 99.2% ( = 100–5/6) CIs.

^β^Adjusted for Sex, Birth conditions, Parental education level, Annual household income, Postnatal feeding method, Lactose intolerance, Regular calcium supplementation, Regular vitamin D supplementation, Fresh milk intake, Cheese intake/ Yogurt intake, Red meat intake, Processed meat, Snack intake, Sugar-sweetened beverages intake, Frequency of exercise, Bedtime per night at the baseline/Bedtime per night at the 3-month follow-up/ Bedtime per night at the 6-month follow-up.

^δ^Values were multiplied by 6 so that the significance level after Bonferroni correction remains at 5%.

The *post-hoc* power analysis showed that, under the assumption that the estimates from the adjusted model were the true population parameters, the current sample size could provide 23.9% power to detect an effect where the upper limit of the corrected OR confidence interval fell below 1. Furthermore, it provided 34.5% power to detect a statistically significant result in the trend test, with a corrected significance level set at 0.05/6. The finding of low *post-hoc* power suggested that the study was likely underpowered. Given the limited sample size, the effect estimates should be interpreted with caution. Our findings primarily suggest a trend that warrants verification in larger, adequately powered studies.

## Discussion

The present prospective cohort study investigated the association between the baseline intake of cheese and yogurt and sleep duration in preschool-aged children at three different time points. Based on current some studies, the possible mechanisms of cheese and yogurt affecting sleep are as follows.

Cheese and yogurt are dairy products rich in bioavailable nutrients such as tryptophan, calcium, and casein-derived bioactive peptides. Dairy products are notably rich in tryptophan (Trp), a key substrate for serotonin and melatonin production, which are instrumental for initiating and maintaining sleep, dairy products provide a range of micronutrients that serve as cofactors in the synthesis of melatonin from Trp, which could contribute to sleep-promoting effects (22). Dairy-derived bioactive peptides, such as casein hydrolysates, have been shown to possess anxiolytic and sedative properties in animal studies, the underlying mechanisms may involve the regulation of the GABAergic system, tryptophan metabolism, and the cAMP signaling pathway (23, 24), the release of specific bioactive peptides (e.g., newly identified tetrapeptides), the promotion of sleep via modulation of neuronal electrophysiological activity (25), and the influence on tryptophan metabolic pathways through the gut–brain axis, thereby regulating sleep-related neurotransmitters such as serotonin (5-HT) and dopamine (24, 26). Dairy fat, especially unsaturated fatty acids, may modulate sleep-related outcomes (27). Moreover, cheese and yoghurt are both foods that contain probiotics, and modulation of the intestinal microbiota has been shown to improve sleep through the modulation neurotransmitters (13, 28), cytokine levels (29) and circadian gene expression (30).

Our findings indicated that cheese intake of ≥ 7 servings/week was significantly associated with lower odds of insufficient sleep at the 6-month follow-up. although the specific microbial profiles of the cheese products consumed by our participants were not assessed, it is conceivable that frequent cheese intake may influence microbial diversity and metabolite production, thereby promoting better sleep. However, no significant association was observed between yogurt intake and sleep duration at any of the three time points. This contrasts with findings from adult studies (14, 15, 22, 31), where yogurt consumption was linked to improved sleep quality and reduced risk of sleep disorders. One possible explanation is the differential composition of probiotics and prebiotics between cheese and yogurt. Cheese undergoes a longer fermentation and aging process (especially natural cheese), which may foster a more diverse and stable microbial community, including strains with stronger psychobiotic potential, while some commercial yogurts may contain lower amounts of live cultures. Additionally, the higher fat content (especially unsaturated fatty acids) and bioavailability of certain micronutrients in cheese may synergistically enhance the bioavailability of sleep-promoting compounds (22, 27), It is also plausible that children who consume cheese frequently may have other unmeasured lifestyle or dietary habits that contribute to better sleep hygiene.

Moreover, an association between consuming cheese ≥ 7 servings/week and reduced risk of insufficient sleep duration was evident at 6 months but not at baseline or 3 months, these results may suggest a potential cumulative or delayed effect of cheese intake on sleep duration, studies reported that probiotics may need to be consumed continuously to exert their effects, through long-term modulation of the host microbiota and its metabolites (26, 32), the underlying mechanisms and generalizability of these findings warrant further investigation.

To our knowledge, this is the first prospective design study to investigate the relationship between cheese and yogurt intake and sleep duration among preschool-aged children. Moreover, we excluded potential confounding factors, making the results more reliable. Despite these novel insights, several limitations must be acknowledged. Firstly, dietary intake and sleep were assessed using parent-reported questionnaires, which are vulnerable to recall and social desirability biases, however, these methods are widely used in large cohort studies and still have practical value for capturing habitual intake in children. Secondly, cheese and yogurt intake were assessed a questionnaire and it was not possible to compare the specific amounts of probiotics contained in the two dairy products. Thirdly, despite adjusting for a wide range of potential confounders, the possibility of residual confounding cannot be excluded, for example, higher cheese intake may come from families with a stronger health consciousness, and all of these may independently affect sleep duration. Fourthly, missing data accounted for 51%, attrition may have introduced selection bias. However, we compared the baseline characteristics of the complete and missing children and found differences in annual household income, postnatal feeding method, snack intake, but no significant differences in sleep duration, yogurt intake and cheese intake, mitigating concerns about differential attrition. Finally, the sample may be insufficient, particularly cheese intake ≥ 7 servings/week (7 controls, 3 cases). Post hoc power calculations indicated limited statistical power, which constrains precision and the robustness of inference. However, the consistency between the main logistic regression model and the mixed effects model strengthens credibility of the observed trends. A mixed-effects model that considered the correlation between subjects at different time points confirmed a significant interaction between high cheese intake and the 6-month time, strengthening the evidence for potential long-term benefits.

## Conclusion

This study provides preliminary evidence that frequent cheese intake may be associated with a reduced risk of insufficient sleep duration in preschool-aged children over a 6-month period. While no such association was found for yogurt, the differential results highlight the need for product-specific analyses in future studies. Despite limitations related to sample size and measurement, our findings contribute to the growing body of literature on the gut-brain axis and its implications for child health. Further investigation is warranted to elucidate the mechanisms underlying these associations and to translate these insights into actionable dietary recommendations.

## Data Availability

The raw data supporting the conclusions of this article will be made available by the authors, without undue reservation.

## References

[1] Balog P Tesch D Poós A. Background factors of childhood sleep disorders: interparental conflicts, parent-child attachment, parenting style and the quality of parent-child relationship. *Orv Hetil.* (2024) 165:652–63. 10.1556/650.2024.33016 38678553

[2] Liu H Ma S Feng L Gao J Wu B Xia W Longitudinal association of nighttime sleep duration with emotional and behavioral problems among rural preschool children. *Eur Child Adolesc Psychiatry.* (2024) 33:267–77. 10.1007/s00787-023-02153-4 36781466 PMC9925221

[3] van Tetering E Mies G Klip H Pillen S Muskens J Polderman T The relationship between sleep difficulties and externalizing and internalizing problems in children and adolescents with mental illness. *J Sleep Res.* (2025) 34:e14398. 10.1111/jsr.14398 39533513 PMC12069754

[4] Goetz A Jindal I Moreno J Puyau M Adolph A Musaad S The roles of sleep and eating patterns in adiposity gain among preschool-aged children. *Am J Clin Nutr.* (2022) 116:1334–42. 10.1093/ajcn/nqac197 35833269 PMC9630867

[5] Duraccio K Xu Y Beebe D Lanphear B Chen A Braun J High levels of sleep disturbance across early childhood increases cardiometabolic disease risk index in early adolescence: longitudinal sleep analysis using the health outcomes and measures of the environment study. *Sleep.* (2024) 47:zsad318. 10.1093/sleep/zsad318 38092369 PMC10925946

[6] Yin Q Liu C Bao H Li S Huang Z Gu D Estimation of gingival crevicular fluid oxidative stress markers in school-aged children and teenagers with insufficient sleep. *BMC Oral Health.* (2022) 22:616. 10.1186/s12903-022-02642-z 36529715 PMC9759888

[7] Sujaya I Mariyatun M Hasan P Manurung N Pramesi P Juffrie M Randomized study of *Lacticaseibacillus* fermented milk in Indonesian elderly houses: impact on gut microbiota and gut environment. *World J Gastroenterol.* (2025) 31:104081. 10.3748/wjg.v31.i12.104081 40182598 PMC11962840

[8] Ma J Wang J Wan Y Wang S Jiang C. Probiotic-fermented traditional Chinese herbal medicine, a promising approach to maintaining the intestinal microecology. *J Ethnopharmacol.* (2025) 337:118815. 10.1016/j.jep.2024.118815 39270882

[9] Shen X Jin H Zhao F Kwok L Zhao Z Sun Z. Short-term probiotic supplementation affects the diversity, genetics, growth, and interactions of the native gut microbiome. *Imeta.* (2024) 3:e253. 10.1002/imt2.253 39742297 PMC11683461

[10] Yang H Liu Z Jin Y Liu Z Zhang B Yuan Z Preventive and reparative functions of host-associated probiotics against soybean meal induced growth, immune suppression and gut injury in Japanese seabass (Lateolabraxjaponicus). *Fish Shellfish Immunol.* (2022) 128:651–63. 10.1016/j.fsi.2022.08.034 36028056

[11] Sivamaruthi B Chaiyasut C Sisubalan N Kesika P. The impact of probiotic supplementation on the sleep quality of humans: a review of results of randomized, blinded, controlled studies. *Curr Pharm Des.* (2025) 31:3128–38. 10.2174/0113816128370349250413163229 40337962

[12] Sejbuk M Siebieszuk A Witkowska A. The role of gut microbiome in sleep quality and health: dietary strategies for microbiota support. *Nutrients.* (2024) 16:2259. 10.3390/nu16142259 39064702 PMC11279861

[13] Tang M Song X Zhong W Xie Y Liu Y Zhang X. Dietary fiber ameliorates sleep disturbance connected to the gut-brain axis. *Food Funct.* (2022) 13:12011–20. 10.1039/d2fo01178f 36373848

[14] Liu Y Chen Y Zhang Q Zhang Y Xu F. A double blinded randomized placebo trial of Bifidobacterium animalis subsp. lactis BLa80 on sleep quality and gut microbiota in healthy adults. *Sci Rep.* (2025) 15:11095. 10.1038/s41598-025-95208-2 40169760 PMC11961682

[15] Yang R Lin S Xie X Tang Y Zheng J Yuan C Association between yogurt and dietary supplements containing probiotic consumption with sleep disturbance in US adults: results from NHANES, 2009-2018. *Heliyon.* (2024) 10:e35609. 10.1016/j.heliyon.2024.e35609 39170211 PMC11336832

[16] Holzhausen E Peppard P Sethi A Safdar N Malecki K Schultz A Associations of gut microbiome richness and diversity with objective and subjective sleep measures in a population sample. *Sleep.* (2024) 47:zsad300. 10.1093/sleep/zsad300 37988614 PMC10926107

[17] Carpena M Barros A Comelli E López-Domínguez L Alves ED Wendt A Accelerometer-based sleep metrics and gut microbiota during adolescence: association findings from a Brazilian population-based birth cohort. *Sleep Med.* (2024) 114:203–9. 10.1016/j.sleep.2023.12.028 38219656

[18] Sugimori N Hamazaki K Matsumura K Kasamatsu H Tsuchida A Inadera H. Association between maternal fermented food consumption and infant sleep duration: the Japan Environment and children’s study. *PLoS One.* (2019) 14:e0222792. 10.1371/journal.pone.0222792 31584958 PMC6777830

[19] Inoue M Sugimori N Hamazaki K Matsumura K Tsuchida A Inadera H. Association between maternal fermented food consumption and child sleep duration at the age of 3 years: the Japan environment and children’s study. *BMC Public Health.* (2022) 22:1504. 10.1186/s12889-022-13805-6 35933371 PMC9356427

[20] Inoue M Sugimori N Hamazaki K Matsumura K Tsuchida A Inadera H. Dietary intake of yogurt and cheese in children at age 1 year and sleep duration at age 1 and 3 years: the Japan environment and children’s study. *BMC Pediatr.* (2022) 22:624. 10.1186/s12887-022-03633-3 36319988 PMC9623995

[21] Hirshkowitz M Whiton K Albert S Alessi C Bruni O DonCarlos L National Sleep Foundation’s updated sleep duration recommendations: final report. *Sleep Health.* (2015) 1:233–43. 10.1016/j.sleh.2015.10.004 29073398

[22] St-Onge M Zuraikat F Neilson M. Exploring the role of dairy products in sleep quality: from population studies to mechanistic evaluations. *Adv Nutr.* (2023) 14:283–94. 10.1016/j.advnut.2023.01.004 36774251 PMC10229376

[23] Qian J Zheng L Hong Z Zhao M. Metabolomic analysis reveals the linkage between sleep-enhancing effects and metabolite biomarkers and pathways of different casein hydrolysates in chronic unpredictable mild stressed mice. *J Agric Food Chem.* (2024) 72:25675–89. 10.1021/acs.jafc.4c07140 39501924

[24] Joung J Song J Kim H Oh N. Protective effects of milk casein on the brain function and behavior in a mouse model of chronic stress. *J Agric Food Chem.* (2021) 69:1936–41. 10.1021/acs.jafc.0c07292 33496183

[25] Chen Y Xu L Lan Y Liang C Liu X Li J Four novel sleep-promoting peptides screened and identified from bovine casein hydrolysates using a patch-clamp model in vitro and Caenorhabditis elegans in vivo. *Food Funct.* (2023) 14:6142–56. 10.1039/d3fo01246h 37334648

[26] Li P Yang L Shao X Zou Z Shi H Sun Y Lactobacillales derived from traditional Xizang dairy products improve insomnia and restore neurotransmitter-metabolic profiles via gut microbiota in PCPA-induced mice. *Microbiol Res.* (2025) 8:128276. 10.1016/j.micres.2025.128276 40645156

[27] Zhuang P Wu Y Yao J Liu X Liu H Wan X Marine n-3 polyunsaturated fatty acids slow sleep impairment progression by regulating central circadian rhythms in type 2 diabetes. *Cell Rep Med.* (2025) 6:102128. 10.1016/j.xcrm.2025.102128 40347940 PMC12147914

[28] Cheng H Yang W Xu H Zhu W Gong A Yang X Microbiota metabolites affect sleep as drivers of brain gut communication (Review). *Int J Mol Med.* (2025) 56:130. 10.3892/ijmm.2025.5571 40613226 PMC12236747

[29] Qian X Song X Liu X Chen S Tang H. Inflammatory pathways in Alzheimer’s disease mediated by gut microbiota. *Ageing Res Rev.* (2021) 68:101317. 10.1016/j.arr.2021.101317 33711509

[30] Wang Z Wang Z Lu T Yuan G Chen W Jin J Gut microbiota regulate insomnia-like behaviors via gut-brain metabolic axis. *Mol Psychiatry.* (2025) 30:2597–611. 10.1038/s41380-024-02867-0 39658705

[31] Kinoshita T Maruyama K Suyama K Nishijima M Akamatsu K Jogamoto A Consumption of OLL1073R-1 yogurt improves psychological quality of life in women healthcare workers: secondary analysis of a randomized controlled trial. *BMC Gastroenterol.* (2021) 21:237. 10.1186/s12876-021-01793-7 34030638 PMC8142513

[32] WuQ GaoG KwokL LvH SunZ. Insomnia: the gut microbiome connection, prospects for probiotic and postbiotic therapies, and future directions. *J Adv Res.* (2025) 10:5. 10.1016/j.jare.2025.07.005 40651630

