# Supplementary material for: Association of cheese and yogurt intake with sleep duration in preschool-aged children: a 6-month prospective cohort study
Source: Front Nutr. 2026 Jan 5;12:1685564. doi: 10.3389/fnut.2025.1685564 (PMC12813419; doi:10.3389/fnut.2025.1685564)
Supplement: Supplementary file 1 [file Table_1.pdf]

TABLE S1: Comparison of baseline characteristics between cases with complete and missing data

Characteristics	Complete (N=221)	Missing (N=233)	p-value
**Sex**			0.895
Boy	121 (54.8%)	129 (55.4%)	
Girl	100 (45.2%)	104 (44.6%)	
**Birth conditions**			0.578
Preterm infants	19 (8.6%)	13 (5.6%)	
Term small for gestational age infants	8 (3.6%)	7 (3%)	
Term normal birth weight infants	193 (87.3%)	211 (90.6%)	
Large for gestational age infants	1 (0.5%)	2 (0.9%)	
**Parental education level**			0.115
High school or below	42 (19%)	60 (25.8%)	
University	170 (76.9%)	159 (68.2%)	
Graduate school or above	9 (4.1%)	14 (6%)	
**Annual household income (CNY)**			**0.011**
≤8000	44 (19.9%)	76 (32.6%)	
8001-15000	115 (52%)	92 (39.5%)	
15001-30000	47 (21.3%)	47 (20.2%)	
≥30001	15 (6.8%)	18 (7.7%)	
**Postnatal feeding method**			**0.048**
≥6 months exclusive breastfeeding	121 (54.8%)	113 (48.5%)	
<6 months exclusive breastfeeding	26 (11.8%)	42 (18%)	
Mixed feeding	60 (27.1%)	52 (22.3%)	
Exclusive formula feeding	14 (6.3%)	26 (11.2%)	
**Lactose intolerance**			1

No	216 (97.7%)	228 (97.9%)	
Yes*	5 (2.3%)	5 (2.1%)	
**Regular calcium supplementation**			0.327
No	179 (81%)	180 (77.3%)	
Yes	42 (19%)	53 (22.7%)	
**Regular vitamin D supplementation**			0.088
No	150 (67.9%)	175 (75.1%)	
Yes	71 (32.1%)	58 (24.9%)	
**Fresh milk intake**			0.066
Never	15 (6.8%)	34 (14.6%)	
1 serving/week	33 (14.9%)	33 (14.2%)	
2-6 servings/week	63 (28.5%)	60 (25.8%)	
≥7 servings/week	110 (49.8%)	106 (45.5%)	
**Yogurt intake**			0.172
Never	31 (14%)	51 (21.9%)	
1 serving/week	94 (42.5%)	89 (38.2%)	
2-6 servings/week	66 (29.9%)	67 (28.8%)	
≥7 servings/week	30 (13.6%)	26 (11.2%)	
**Cheese intake**			0.115
Never	74 (33.5%)	103 (44.2%)	
1 serving/week	108 (48.9%)	94 (40.3%)	
2-6 servings/week	29 (13.1%)	29 (12.4%)	
≥7 servings/week	10 (4.5%)	7 (3%)	
**Red meat intake#**			0.431
Never	30 (13.6%)	40 (17.2%)	
1 serving/week	71 (32.1%)	81 (34.8%)	

2-6 servings/week	74 (33.5%)	75 (32.2%)	
≥7 servings/week	46 (20.8%)	37 (15.9%)	
**Processed meat[&]**			0.493
Never	126 (57%)	130 (55.8%)	
1 serving/week	78 (35.3%)	83 (35.6%)	
2-6 servings/week	12 (5.4%)	18 (7.7%)	
≥7 servings/week	5 (2.3%)	2 (0.9%)	
**Snack intake** [Δ]			**0.003**
Never	20 (9%)	50 (21.5%)	
1 serving/week	116 (52.5%)	105 (45.1%)	
2-6 servings/week	72 (32.6%)	64 (27.5%)	
≥7 servings/week	13 (5.9%)	14 (6%)	
**Sugar-sweetened beverages intake**			0.498
Never	77 (34.8%)	90 (38.6%)	
1 serving/week	114 (51.6%)	107 (45.9%)	
2-6 servings/week	28 (12.7%)	31 (13.3%)	
≥7 servings/week	2 (0.9%)	5 (2.1%)	
**Frequency of exercise**			0.149
0-1 time/week	12 (5.4%)	21 (9%)	
2-3 times/week	65 (29.4%)	83 (35.6%)	
4-5 times/week	80 (36.2%)	75 (32.2%)	
6-7 times/week	64 (29%)	54 (23.2%)	
**Bedtime each night**			0.189
Before 9:00pm	17 (7.7%)	29 (12.4%)	
9:00-10:00pm	115 (52%)	114 (48.9%)	
10:00-11:00pm	82 (37.1%)	77 (33%)	

After 11:00pm	7 (3.2%)	13 (5.6%)	
**Sleep time/day**			0.551
≥10h	120 (54.3%)	133 (57.1%)	
<10h	101 (45.7%)	100 (42.9%)	

CNY, Chinese Yuan

*unable to drink milk on an empty stomach, but can consume yogurt or cheese.

[#] Red meat, such as pork, beef, lamb, liver, blood products, etc.

[&]Red sausage, sausage, bacon, smoked meat, etc.

Δcandy, jelly, ice cream, cake, cookies, chocolate, potato chips, etc.

Bold indicates significance

TABLE S2: Results of the mixed-effects logistic regression model with random intercepts about the associations between cheese intake and yogurt intake and sleep time at the baseline, 3-month follow-up and 6-month follow-up.

Fixed effects	OR (95% CI)	p-value
Time (3 month)	2.673 (1.415, 5.048)	0.002
Time (6 month)	5.145 (2.618, 10.111)	<0.001
High intake of yogurt	0.862 (0.357, 2.078)	0.740
High intake of cheese stick	1.627 (0.516, 5.127)	0.406
High intake of yogurt - Time (3 month)	1.714 (0.596, 4.925)	0.317
High intake of yogurt - Time (6 month)	2.797 (0.892, 8.771)	0.078
High intake of cheese stick - Time (3 month)	0.324 (0.083, 1.270)	0.106
High intake of cheese stick - Time (6 month)	0.217 (0.052, 0.917)	**0.038**
Variance of random effects	2.683	
p-value for likelihood ratio test comparing mixed-effects model and fixed-effects model	<0.001	

Bold indicates significance

OR (95% CI): Odds ratios (95% confidence interval)

To mitigate potential model convergence issues due to model complexity, we dichotomized the intake variables. Specifically, “never” and “1 servings/week” were classified as low intake, while “2-6 servings/week” and “≥7 servings/week” were classified as high intake.
